# Modeling Short-Term Maximum Individual Exposure from Airborne Hazardous Releases in Urban Environments. Part I: Validation of a Deterministic Model with Field Experimental Data

**DOI:** 10.3390/toxics3030249

**Published:** 2015-06-25

**Authors:** George C. Efthimiou, John G. Bartzis, Michail Palaiokostas

**Affiliations:** 1 Environmental Research Laboratory, INRASTES, NCSR Demokritos, Patriarchou Grigoriou & Neapoleos Str., 15310 Aghia Paraskevi, Greece; 2 Department of Mechanical Engineering, University of Western Macedonia, Sialvera & Bakola Str., 50100 Kozani, Greece; E-Mail: bartzis@uowm.gr; 3 School of Engineering and Materials Science, Queen Mary University of London, Mile End Road, E1 4NS London, UK; E-Mail: m.palaiokostas@qmul.ac.uk

**Keywords:** maximum individual exposure, field measurements, turbulence integral time scale, model optimization

## Abstract

The release of airborne hazardous substances in the atmosphere has a direct effect on human health as, during the inhalation, an amount of concentration is inserted through the respiratory system into the human body, which can cause serious or even irreparable damage in health. One of the key problems in such cases is the prediction of the maximum individual exposure. Current state of the art methods, which are based on the concentration cumulative distribution function and require the knowledge of the concentration variance and the intermittency factor, have limitations. Recently, authors proposed a deterministic approach relating maximum individual exposure to parameters such as the fluctuation intensity and the concentration integral time scale. The purpose of the first part of this study is to validate the deterministic approach with the extensive dataset of the MUST (Mock Urban Setting Test) field experiment. This dataset includes 81 trials, which practically cover various atmospheric conditions and stability classes and contains in total 4004 non-zero concentration sensor data with time resolutions of 0.01–0.02 s. The results strengthen the usefulness of the deterministic model in predicting short-term maximum individual exposure. Another important output is the estimation of the methodology uncertainty involved.

## 1. Introduction

One of the key problems in coping with deliberate or accidental atmospheric releases of hazardous materials is the ability to reliably predict not only the concentration levels but also the individual exposure for a given time interval. This is justified by the fact that the release of hazardous substances in the atmosphere has a direct effect on human health as, during inhalation, an amount of concentration is inserted through the respiratory system into the human body, which can cause serious or even irreparable damage in health. The effects on the human health do not only depend on the amount of concentration of the released substance, but also on the time that a person is exposed to high concentration (usually the time is of the order of magnitude of a few seconds or minutes). Therefore, the individual exposure is defined as the amount of concentration inhaled at an exposure time interval, Δτ:

(1) $$D(\Delta \tau )=\int_{\Delta \tau }C(t)\;dt$$

where *D(*Δτ*)* is the individual exposure (μg s m^−3^) at a time interval Δτ [s], and *C(t)* is the instantaneous concentration (μg m^−3^).

The high turbulence inside the urban complexity as well as the low winds has a direct effect on the individual exposure prediction (e.g., [1]). The stochastic nature of turbulence is perceivable during the analysis of experimental concentration time series where zero concentration intervals appear. The urban network causes the natural variability of the wind conditions due to the local turbulence and therefore the plume follows different trajectories for different release times [2]. In other words, how the dispersion of the plume will evolve depends on what are the instantaneous local atmospheric boundary conditions during the release. However, these conditions cannot be defined practically. Therefore the actual concentration and the actual individual exposure at a point downstream from the source are practically unknown quantities. It is more realistic to consider that the actual exposure lies inside a range of values, within which the maximum value is expected to be. The maximum exposure value is the most important parameter for human health and can be defined as the expected maximum individual exposure. Therefore, for the assessment of consequences and countermeasures, it is more realistic to predict the expected maximum individual exposure at a given time interval rather than the actual individual exposure. The expected maximum individual exposure at a given time interval, Δτ, is defined as follows:

(2) $$D_{\text{max}}(\Delta \tau )={\left[\int_{\Delta \tau }C(t)\;dt\right]}_{\text{max}}=C_{\text{max}}\left(\Delta \tau \right)\;\Delta \tau$$

where *C_max_(*Δτ*)* is the maximum time averaged concentration at the interval Δτ.

In summary, the real problem in the present work is set as follows: A hazardous air pollutant is released from a point source. The release can be instantaneous, continuous or finite and is characterized by the maximum release rate at the source. The question is whether the expected maximum individual exposure, *D_max_(*Δτ*)*, can be predicted at a given point downwind from the source.

This study is divided in two parts. The first part presents the models that are used mostly for the prediction of the expected short-term maximum individual exposure. Then a deterministic model is presented, it is optimized with respect to its parameters and it is validated against field experimental data at the measurement time intervals. In the second part of the study, the same deterministic model is validated against wind tunnel experimental data. In this case, an optimization of the model is performed, also with respect to its parameters as well as its performance for a wide range of time intervals.

## 2. Short-Term Maximum Individual Exposure Models

### 2.1. Probabilistic Models

The usual methodology to predict the expected short-term maximum individual exposure *D_max_(*Δτ*)* is based on the use of theoretical statistical distributions. Concentration parameters such as the mean, $\bar{C}$, the variance, $\bar{{C^{\prime }}^{2}}$, and the intermittency factor, *γ* (defined as the ratio of the number of concentration zero data to the total number of data), are introduced in a selected, known cumulative distribution function and by using a confidence limit (e.g. 95% or 99%) or a predetermined value (threshold), the expected peak concentration is predicted and therefore the maximum individual exposure, according to Equation (2), for any time interval.

Some of the basic statistical distributions that have been examined in the literature are the Gamma distribution (e.g. [3,4,5,6]), the Lognormal distribution ([3,4,5,7]), the Weibull distribution ([3,4]), the Exponential distribution ([4,6,7,8]), the Chopped normal distribution ([4,6,7,8]) and the Generalized Pareto distribution [9].

This methodology presents some limitations. There is not a common statistical distribution that can be used in all cases and usually at least two theoretical distributions are examined in order to find the one that presents the best fit with the experimental concentration data. Additionally, most of the theoretical distributions do not conclude to a finite maximum value. As a result, it becomes imperative to use confidence intervals, for example 99% or 95%. Finally, the probabilistic methodology is focused on the prediction of the instantaneous concentration. Mylne and Mason (1991) [8] have shown during the analysis of experimental data that different time intervals can distort the concentration cumulative distribution function, a fact that concludes to different maximum individual exposure values.

### 2.2. Deterministic Models

On the other hand, the maximum individual exposure is expected to be finite and the use of a deterministic model is more attractive. A simple deterministic equation can be of the form [10,11]:

(3) $$\frac{C_{\text{max}}\left(\Delta \tau \right)}{{\text{C}}_{\text{max}}\left(\Delta Τ\right)}={\left(\frac{\Delta \tau }{\Delta Τ}\right)}^{-n}$$

where *C_max_(*Δτ*)* is the unknown expected maximum concentration at any time interval Δτ, *C_max_(*Δ*T)* is the reference maximum concentration at the time interval ΔΤ (e.g., one hour), and *n* is an exponent that can be estimated experimentally.

A deterministic model based on Equation (3) has been presented in Bartzis *et al.* (2008) [12] where the maximum individual exposure is given as a function of the fluctuation intensity (*I*) and the turbulence autocorrelation time scale (*T_C_*):

(4) $$D_{\text{max}}\left(\Delta \tau \right)=\bar{C}\;\left[1+\beta \;Ι\;{\left(\frac{\Delta \tau }{T_{C}}\right)}^{-n}\right]\;\Delta \tau$$

The turbulence autocorrelation time scale is estimated as follows:

(5) $$T_{C}=\int_{0}^{\infty }R_{C}(\tau )d\tau$$

where *R_C_(*τ*)* is the autocorrelation coefficient.

The fluctuation intensity is defined as:

(6) $$I=\frac{\bar{{C^{\prime }}^{2}}}{{\bar{\text{C}}}^{2}}$$

In Equation (4), the parameters *β* and *n* are estimated experimentally. The stochastic nature of turbulence in combination with the finite sampling intervals of a concentration time series does not allow these parameters to take precise values. A first effort to estimate these parameters was performed in the same study [12]. The representative values under neutral stability conditions are:

(7) β=1.5,n=0.3

The number of the experimental data used for the derivation of the above values, Equation (7), was limited and the evaluation was restricted only for neutral stability conditions. In order to use the model for risk assessment studies, it should be evaluated with experimental data of various stability classes and the concentration database of the MUST (Mock Urban Setting Test) experiment [13] is ideal for this purpose.

## 3. The Concentration Database of the MUST Experiment

The concentration database of the MUST experiment includes 81 trials. The total number of concentration sensor data for all the trials is 5832, from which 3888 sensor data have a measurement time interval Δτ = 0.01 s and 1944 sensor data have Δτ = 0.02 s. The database covers mainly stable and neutral stability classes [13]. In the present study all the available concentration data are used.

### Evaluation and Manipulation of the Concentration Data

The evaluation of the concentration data was performed by Yee and Biltoft (2004) [13], including control for possible measurement errors and control of the unsteadiness that is inherent in the real meteorological data. A MATLAB [14] code was developed in this work for data manipulation. Totally, 1.36 × 10^8^ data points were manipulated. The following variables were estimated from each time series: the maximum concentration *C_max_(*Δτ*)*, the mean concentration $\bar{C}$, the concentration variance $\bar{{C^{\prime }}^{2}}$, and the autocorrelation time scale of concentration *Τ_C_*.

## 4. Refinement of Model’s Parameters

The parameters *β* and *n* of Equation (4) present considerable variability when they are estimated from experimental data. This is based mainly on the imperfections of the model, the partial unsteadiness of the concentration time series, as well as on the fact that the measurement signals are often governed by non-local large-scale disturbances.

In order to make the process of estimating the uncertainty of the model’s parameters simple and functional, the following steps are applied:

1. The proportionality constant *β* is calculated by keeping the exponent *n* constant and equal to the representative value (*n* = 0.3). The proportionality constant *β* is calculated as follows:
  (8) $$\beta =\frac{\frac{C_{\text{max}}(\Delta \tau )-\bar{C}}{\bar{C}}}{I\;{\left(\frac{\Delta \tau }{T_{C}}\right)}^{-n}}$$
2. The exponent *n* is calculated by keeping the parameter *β* constant and equal to the representative value (*β* = 1.5). The exponent *n* is calculated as follows:
  (9) $$n=-\frac{\text{ln}\left[\frac{C_{\text{max}}(\Delta \tau )-\bar{C}}{\bar{C}\beta \;Ι}\right]}{\text{ln}\;\left[\;\frac{\Delta \tau }{T_{C}}\right]}$$

By following this strategy, it is expected that the imperfectness of Equation (4) as well as the possible measurement errors will be reflected at the values of *β* and *n* and in their variability/uncertainty.

Table 1 presents the maximum, mean and standard deviation values of the parameters *β* and *n*. The estimated mean values of the parameters *β* and *n* (1.72 and 0.31) are very close to the representative values (1.5 and 0.3), a fact that supports the robustness of the model. It should also be noted that the maximum value of the parameter *β* directly affects the upper bound of the expected maximum individual exposure, as it is proportionality constant in Equation (4). From Table 1, it is obvious that the maximum *β* value is almost seven times higher than the mean value.

**Table 1:** Statistical values of the parameters *β* and *n*.

Parameters	Maximum	Mean	Standard Deviation
*β*	12.46	1.72	1.18
*n*	9.74	0.31	0.28

### Probability Density Functions of the Parameters

**Figure 1:**
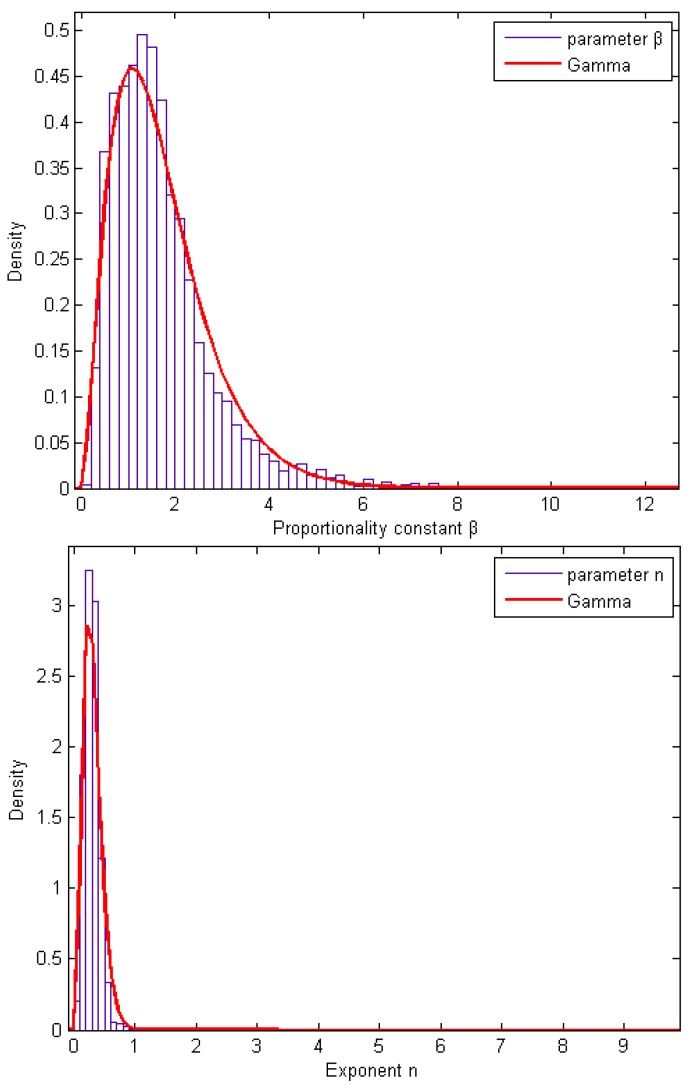
The probability density functions of parameters *β* and *n* and the fit of the Gamma distribution.

Figure 1 presents the probability density functions of the parameters *β* and *n*. For both parameters, the Gamma distribution seems to describe the data adequately.

Table 2 presents the Gamma distribution statistics after their fitting to the *β* and *n* distributions. The probability density function of the Gamma distribution for a variable *x* is given as follows:

(10) $$P_{\Gamma }(x|a,b)=\frac{1}{b^{a}\Gamma (a)}x^{a-1}e^{-x/b}$$

where *a* is the shape parameter, *b* is the scale parameter and *Γ(a)* is the Gamma function, which is estimated as follows:

(11) $$\Gamma (a)=\int_{0}^{\infty }e^{-t}t^{a-1}dt$$

It should be noted that the Gamma fit was performed using the MATLAB software [14] where the Maximum Likelihood Estimation was used for the estimation of the distribution’s parameters, *a* and *b*.

**Table 2:** Parameters *a* and *b* of the Gamma distribution (including the 95% confidence intervals) and the estimated parameters *β* and *n*.

Gamma Distribution Fit	Parameter *β*	Parameter *n*
*a*	2.71 (95% confidence intervals lower: 2.6, upper: 2.83)	4.51 (95% confidence intervals lower: 4.325, upper: 4.708)
*b*	0.63 (95% confidence intervals lower: 0.605, upper: 0.663)	0.069 (95% confidence intervals lower: 0.066, upper: 0.073)
Mean value (*ab*)	1.72	0.31
Standard deviation ($\sqrt{{ab}^{2}}$)	1.04	0.15

The mean values and the standard deviations of the Gamma distribution of both parameters are comparable with the experimental values given in Table 1, a fact that strengthens the Gamma fitting to the parameters.

In summary, Bartzis *et al.* (2008) [12] model can be further improved, assuming that the proportionality constant *β* is modeled by the Gamma distribution with scale parameter *a* = 2.71 and shape parameter *b* = 0.63, which correspond to a mean value equal to $\bar{\beta }=1.72$. The distribution is valid up to the confidence interval 99.99997%. The mean value of the parameter *n* can remain the same as the representative one (=0.3). Taking into account that the experiments are completely different, this result is very important for the reliability of the present model.

## 5. Performance of the Model

**Figure 2:**
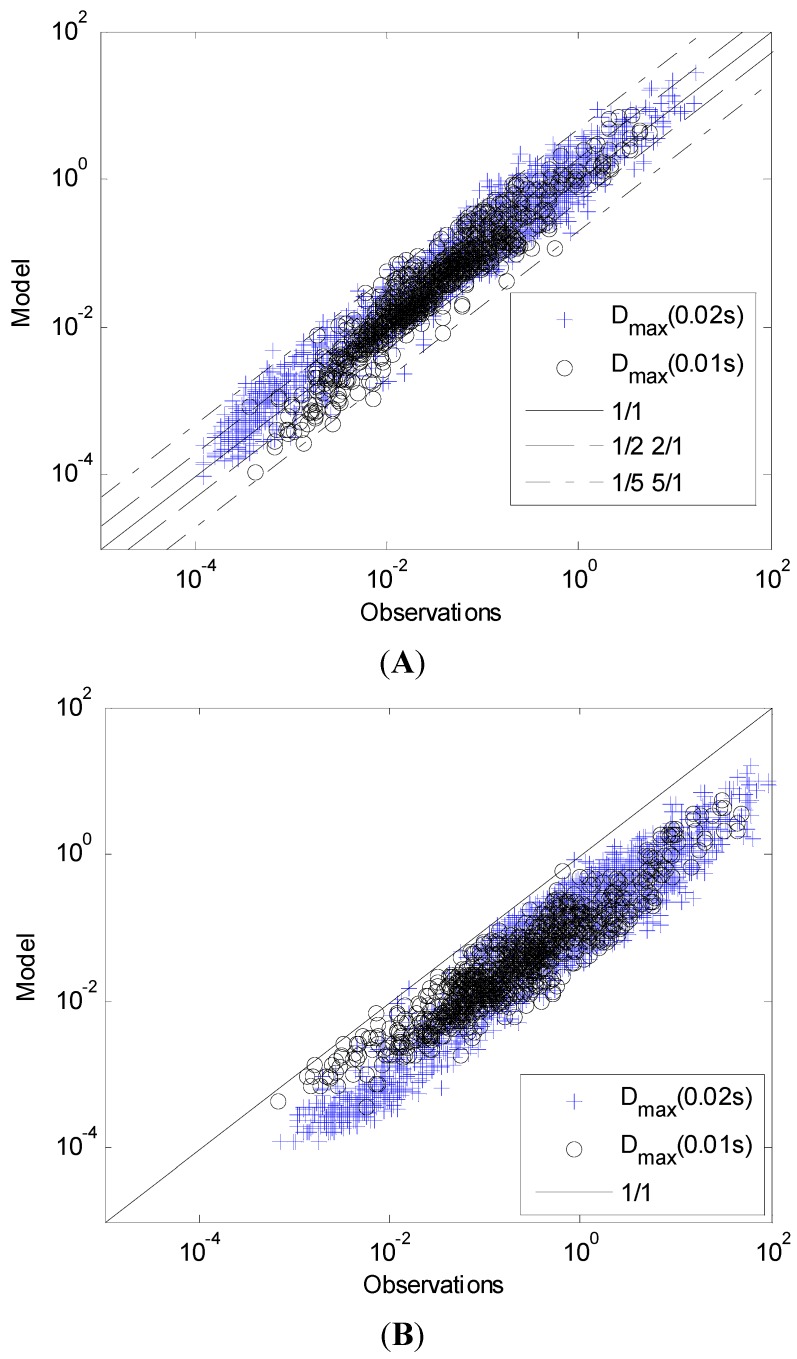
Performance of Bartzis *et al.* (2008) [12] model for *β* = 1.72 (**A**) and *β* = 12.46 (**B**) (Δτ = 0.01–0.02 s).

In Figure 2, the Bartzis *et al.* (2008) [12] model results for *β* = 1.72 and *n* = 0.3 are compared with the experimental measurements. The experimental values of the concentration mean ($\bar{C}$), variance ($\bar{{C^{\prime }}^{2}}$) and integral time scale (*T_C_*) are used in the Bartzis *et al.* (2008) [12] model. The experimental *D_max_(*Δτ*)* values are estimated using Equation (2), i.e. multiplying the maximum concentration of each time series with the corresponding time interval of the instrument. The fraction of predictions within a factor of two, FAC2, and five, FAC5, of observations have been calculated. These two metrics (FAC2 and FAC5) are defined as:

(12) $$\text{FAC}2=\frac{N}{T}=\frac{1}{T}\sum_{i=1}^{T}N_{i}\;\text{where}\;N_{i}\left\{ \begin{array}{l} 1,\;\;\;0.5\leq \frac{C_{\text{pi}}}{C_{\text{oi}}}\leq 2.0\\ 0,\;\;\frac{C_{\text{pi}}}{C_{\text{oi}}}<0.5\;ή\;\frac{C_{\text{pi}}}{C_{\text{oi}}}>2.0 \end{array}\right.$$

(13) $$\text{FAC}5=\frac{N}{T}=\frac{1}{T}\sum_{i=1}^{T}N_{i}\;\text{where}\;N_{i}\left\{ \begin{array}{l} 1,\;\;\;0.2\leq \frac{C_{\text{pi}}}{C_{\text{oi}}}\leq 5.0\\ 0,\;\;\frac{C_{\text{pi}}}{C_{\text{oi}}}<0.2\;ή\;\frac{C_{\text{pi}}}{C_{\text{oi}}}>5.0 \end{array}\right.$$

where *C_pi_* is the prediction, *C_oi_* is the observation/measurement and *T* the total number of data.

The results are very good based on the FAC2 = 80.6%. The FAC5 is equal to 99.2% and this is what is expected, taking into account that *β_max_* = 7 × $\bar{\beta }$. Figure 2 also shows the results for *β* = *β_max_*, where almost all the results are below the ideal line, 1/1. The performance of the model for large Δτ values is examined in Part II of the study.

## 6. Conclusions

The basic research in this study was focused on the prediction of the expected maximum individual exposure in case of accidental or deliberate releases of hazardous substances into the atmosphere.

Bartzis *et al.* (2008) [12] model is simple in its implementation and it overcomes the limitations of the probabilistic models. The basic idea of the model is the correlation of the maximum short-term individual exposure, *D_max_(*Δτ*)*, with the mean value, $\bar{C}$, the fluctuation intensity (*Ι*) and the integral time scale (*T_C_*) (Equation (4)).

For the first time, an extensive concentration database was used for the validation of the model (Equation (4)). More specifically, the concentration database of the MUST experiment [9] was used, which includes 81 trials of various stability classes and includes in total 5832 concentration sensor data with temporal resolutions of 50 and 100 Hz. The analysis revealed a new value for the *β* parameter (=1.72), while the exponent *n* retains the value 0.3. Also for a first time, the variance and the uncertainty of both parameters, *β* and *n*, was systematically studied. Both parameters are well described by the Gamma distribution, with scale and shape coefficients *a* = 2.71 and *b* = 0.63 for the parameter *β* and *a* = 4.51 and *b* = 0.069 for the parameter *n* (the uncertainties of *a* and *b* are also given). The extreme value for the parameter *β* is 7 × 1.72, which corresponds to a probability of 99.99997% of the Gamma distribution. For the new value of the parameter *β* = 1.72, the model *D_max_(*Δτ*)* predicts very well (FAC2 = 80.6%) the maximum individual exposure. The present data analysis highly strengthens the validity of Bartzis *et al.* (2008) [12] model for the prediction of the maximum individual exposure.

It should be noted that more data are important for the evaluation of Bartzis *et al.* (2008) [12] model. In the second part of the study, the same model is validated against wind tunnel experimental data. In this case, an optimization of the model is also performed with respect to its constant parameters as well as its performance for a wide range of time intervals.

## References

[1] Liou P., Vallero D., Foley G., Georgopoulos P., Heiser J., Watson T., Reynolds M., Daloia J., Tong S., Isukapalli S. (2007). A personal exposure study employing scripted activities and paths in conjuction with atmospheric releases of perfluorocarbon tracers in Manhattan, New York. J. Expos. Sci. Environ. Epidem..

[2] Harms F., Hertwig D., Leitl B., Schatzmann M., Patnaik G. Characterization of transient dispersion processes in an urban environment. Proceedings of the 14th Conference on Harmonisation within Atmospheric Dispersion Modelling for Regulatory Purposes.

[3] Lung T., Muller H.J., Glaser M., Moller B. (2002). Measurements and modelling of full-scale concentration fluctuations. Agratechnische Forsch..

[4] Yee E., Kosteniuk P.R., Chandler G.M., Biltoft C.A., Bowers J.F. (1993). Statistical characteristics of concentration fluctuations in dispersing plumes in the atmospheric surface layer. Boundary-Layer Meteorol..

[5] Gailis R.M., Hill A., Yee E., Hilderman T. (2007). Extension of a fluctuating plume model of tracer dispersion to a sheared boundary layer and to a large array of obstacles. Boundary-Layer Meteorol..

[6] Gailis R.M., Hill A. (2006). A Wind-Tunnel Simulation of Plume Dispersion within a Large Array of Obstacles. Boundary-Layer Meteorol..

[7] Yee Ε. (1990). The shape of the probability density function of short-term concentration fluctuations of plumes in the atmospheric boundary layer. Boundary-Layer Meteorol..

[8] Mylne K.R., Mason P.J. (1991). Concentration fluctuation measurements in a dispersing plume at a range of up to 1000 m. Q. J. R. Met. Soc..

[9] Munro R.J., Chatwin P.C., Mole N. (2001). The High Concentration Tails of the Probability Density Function of a Dispersing Scalar in the Atmosphere. Boundary-Layer Meteorol..

[10] Engel P.L., Williams T.O., Muirhead T. Utilizing ISCT to model composting facility odors. Proceedings of the 90th Annual Air and Waste Management Association Meeting and Exhibition.

[11] Naden R.A., Leeds J.V. (1972). The modification of plume models to account for long averaging times. Atmos. Environ..

[12] Bartzis J.G., Sfetsos A., Andronopoulos S. (2008). On the individual exposure from airborne hazardous releases: The effect of atmospheric turbulence. J. Hazard. Mater..

[13] Yee E., Biltoft C.A. (2004). Concentration fluctuation measurements in a plume dispersing through a regular array of obstacles. Boundary-Layer Meteorol..

[14] MATLAB. http://www.mathworks.com/products/matlab/.

